# Structural Dynamics of Protein Interactions Using Site-Directed Spin Labeling of Cysteines to Measure Distances and Rotational Dynamics with EPR Spectroscopy

**DOI:** 10.1007/s00723-023-01623-x

**Published:** 2023-10-11

**Authors:** Osha Roopnarine, David D. Thomas

**Affiliations:** 1 Department of Biochemistry, Molecular Biology and Biophysics, University of Minnesota, Minneapolis, MN 55455, USA

## Abstract

Here we review applications of site-directed spin labeling (SDSL) with engineered cysteines in proteins, to study the structural dynamics of muscle and non-muscle proteins, using and developing the electron paramagnetic resonance (EPR) spectroscopic techniques of dipolar EPR, double electron electron resonance (DEER), saturation transfer EPR (STEPR), and orientation measured by EPR. The SDSL technology pioneered by Wayne Hubbell and collaborators has greatly expanded the use of EPR, including the measurement of distances between spin labels covalently attached to proteins and peptides. The Thomas lab and collaborators have applied these techniques to elucidate dynamic interactions in the myosin–actin complex, myosin-binding protein C, calmodulin, ryanodine receptor, phospholamban, utrophin, dystrophin, β-III-spectrin, and Aurora kinase. The ability to design and engineer cysteines in proteins for site-directed covalent labeling has enabled the use of these powerful EPR techniques to measure distances, while showing that they are complementary with optical spectroscopy measurements.

## 1 Introduction

**Fig. 1:**
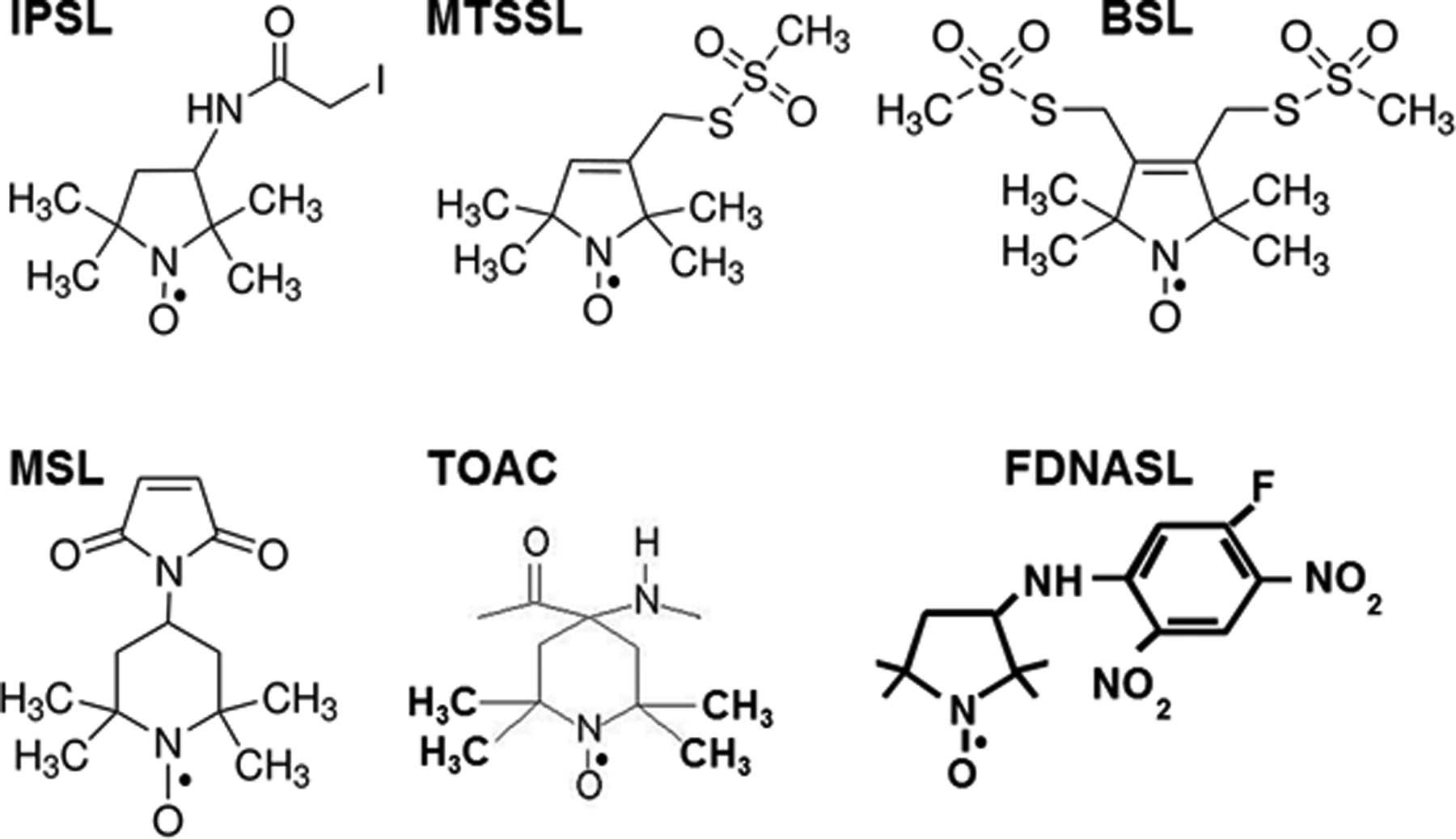
Spin labels used for labeling cysteines in dipolar EPR and DEER measurements. IPSL: 3-(2-iodoacetamido)-PROXL, MTSSL: 1-oxyl-2,2,5,5-tetramethyl-D3-pyrroline-3-methyl methanethio-sulfonate, BSL: 3,4-bis-(methanethio-sulfonylmethyl) - 2,2,5,5-tetramethyl-2,5-dihydro-1H-pyrrol-1-yloxy, MSL: N-(1-oxyl-2,2,5,5-tetramethyl pyrrolidinyl maleimide, TOAC: 2,2,6,6-tetramethyl-piperidine-1-oxyl-4-amino-4-carboxylic acid, FDNASL: 3-(5-fluoro-2,4-dinitroanilino)-PROXYL

Macromolecular assemblies of proteins undergo complex rotational and translational motions that are interdependent. Some proteins form the scaffolds of the assembly, providing stability and anchoring properties, while other proteins are involved in large-scale domain movements that are coupled to smaller movements, all in synchrony to result in contraction of muscle fibers, movement of ions across membranes, movement of domains to allow binding to actin protomers, and transduction of energy from the hydrolysis of ATP to movement. Static structural methodologies that use non-physiological conditions (crystallography and electron microscopy) capture mere snapshots of these dynamic movements that reveal possible models for dynamics, but methodologies such as continuous wave EPR performed under physiological conditions reveal the dynamic nature of protein interactions in the nanosecond to microsecond timescales. The advent of manageable molecular biology techniques for engineering specific cysteines on proteins was the impetus for the development of site-directed spin labeling (SDSL) of proteins, pioneered by Wayne Hubbell [1, 2]. Subsequent adaptation of SDSL with dipolar EPR and DEER enabled distance measurements between judiciously placed spin probes on helices and on the protein backbone, advancing the evolution of EPR spectroscopy technology and complementing distance measurements acquired through fluorescence resonance energy transfer (FRET) spectroscopy. The advantages of EPR over FRET are several fold. (1) The probes used in EPR are smaller, similar to the size of amino acids and are less likely to perturb the structure or function of the protein (Fig. 1). (2) Only one type of probe was required at both sites, in contrast to FRET, which required a probe pair of a donor and acceptor probe. (3) Similarly, EPR can measure rotational dynamics in both the nanosecond (conventional EPR) to microsecond (saturation transfer EPR, developed primarily in the Thomas lab [3–5]) time ranges with a single probe, while optical spectroscopy requires different probes for nanosecond (fluorescent) and microsecond (phosphorescent) time ranges.

In this brief review, we describe selected examples of our own applications of SDSL to protein structure and dynamics over the past four decades, focusing on the past two. We place particular focus on the use of dipolar EPR in to measure distances (8–20 Å) to map the internal structural changes during functionally important binding of ligands and other proteins. We also summarize our use of double electron electron resonance (DEER) to measure longer distances (20–160 Å) using pulsed EPR, and we review selected applications to study orientation and rotational dynamics of spin-labeled proteins. Many of our applications include contractile proteins in muscle, both skeletal and cardiac, both sarcomeric (myosin, calmodulin, phospholamban, myosin-binding protein C) and cytoskeletal (utrophin, dystrophin, β-III-spectrin). We have recently expanded our applications to cancer targets in non-muscle cells (e.g., Aurora kinase).

The basic premise of SDSL is the use of a sulfide-linked nitroxide side chain of a spin label (Fig. 1) for covalent linking with a single cysteine (or double cysteine) to determine secondary structure (backbone structure and dynamics); protein–protein interactions; distances; chain characteristics and depth of a given side chain from the membrane/aqueous surface in membrane proteins; and local electrostatic potentials at solvent-exposed sites [6].

In continuous wavelength (CW) dipolar EPR, the magnetic moment of one spin label creates a local magnetic field (in the presence the applied magnetic field) that influences (or interacts with) the magnetic moment of another proximal spin label. The extent of the dipolar interaction is dependent on the distance, 1/*r*^3^, between the spin labels and the angle, θ, between the magnetic moment of the spin label and the applied magnetic field. Dipolar interaction between the spin labels results in broadening the EPR spectrum (interacting spectrum) compared to the EPR spectrum of the singly labeled sample (noninteracting spectrum).

DEER spectroscopy uses pulsed excitation and time-domain detection to measure the dipolar interaction directly, which results in *sensitivity to longer distances* (20–160 Å) compared to CW dipolar EPR [7]. The combined use of targeted SDSL of proteins with DEER spectroscopy is a powerful combination of techniques to use for discovering structural dynamics within proteins and in protein–protein interactions.

The sensitivity of DEER distance distributions could be diminished by spin label flexibility that could occur with monofunctionally attached spin labels (single cysteine attachment). Hubbell and collaborators developed a stereospecific bifunctional spin label (BSL) with two sites for covalent attachment with cysteines at *i* and *i* + 3 or *i* and *i* + 4 positions on an α-helix or *i* and *i* + 2 on a β-strand to highly constrain the nitroxide side chain [8]. Therefore, for DEER distance measurements, tetra-cysteine mutants for two BSL attachments in the proteins would be required. For rotational mobility measurements such as in ST-EPR and orientation EPR measurements, a di-cysteine mutant for a single BSL would be required.

## 2 Applications of SDSL EPR: Backbone Dynamics

### 2.1 Myosin

**Fig. 2:**
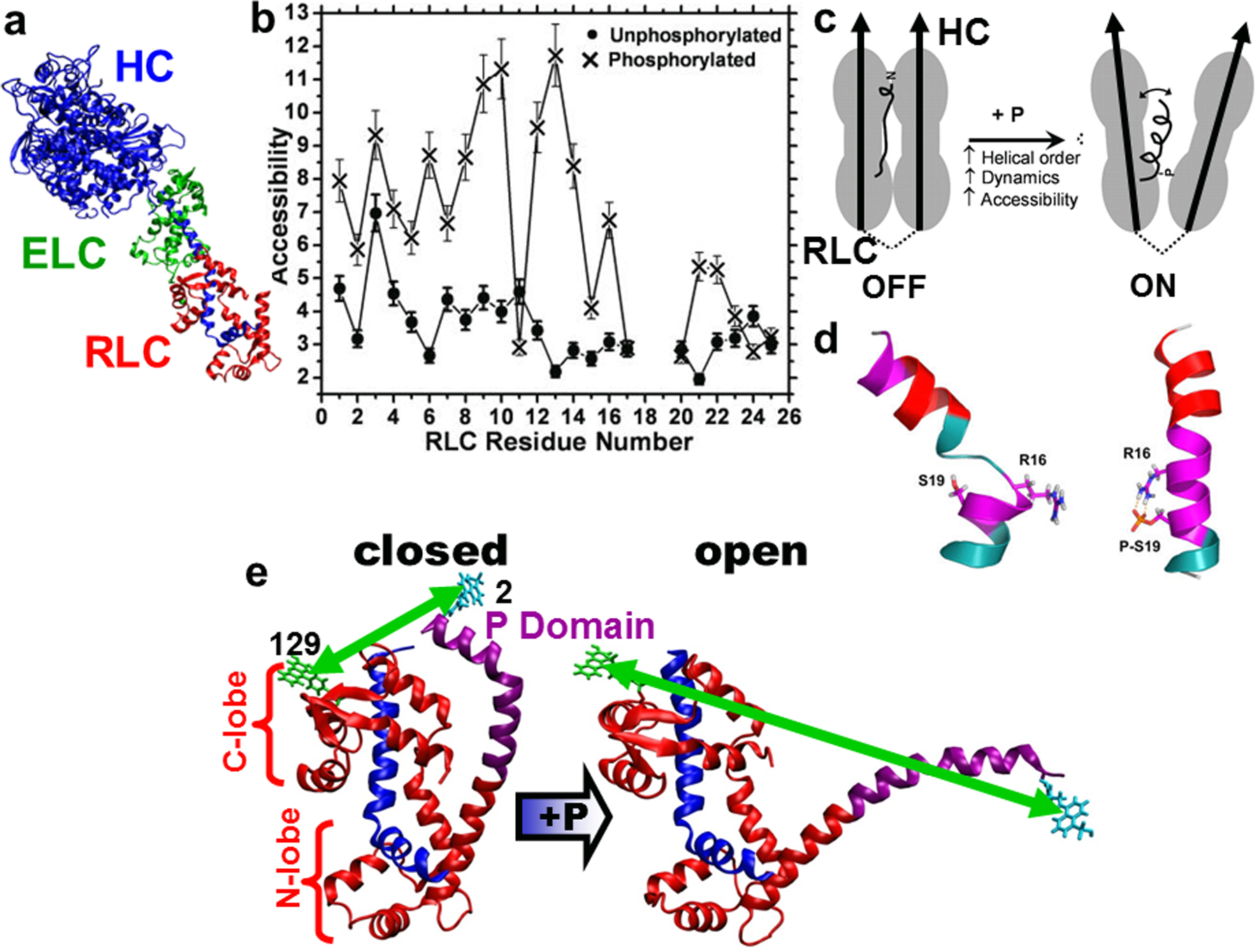
Phosphorylation-induced structural changes in smooth muscle myosin. **a** Myosin head showing the heavy chain (HC, blue), essential light chain (ELC, green) and regulatory light chain (RLC, red). **b** Solvent accessibility of the N-terminus extension (NTE) residues 1–24 of FDNASL-RLC of smooth muscle myosin using dipolar EPR. The RLC-NTE is inaccessible to the solvent in the unphosphorylated state (closed circle) but phosphorylation of RLC renders the NTE solvent accessible (times symbol). **c** Structural model for RLC-NTE (only RLC and a portion of HC is shown) showing that RLC-NTE is mostly disordered and solvent inaccessible in the unphosphorylated state, but is helical and solvent accessible in the phosphorylated state. **d** Molecular dynamics simulation models of RLC-NTE showing that the unphosphorylated NTE has a flexible region preceding R16 that, upon phosphorylation of S19, forms a salt bridge and increases the helicity of the NTE. **e** FRET distance measurements of RLC shows a closed (short distance between probes at Cys 129 and Cys2) conformation in the unphosphorylated RLC and an open (long distance between the probes) upon phosphorylation. Figure is adapted from references with permission [10–13], © (1993, 2005) National Academy of Sciences

Myosin and actin are the main contractile proteins in the sarcomere, each having binding proteins. Myosin is a hexamer consisting of two heavy chains folded into a globular head or catalytic domain (CD) and a long α-helical tail region. The myosin head contains the binding sites for actin and two light chains (LC) (essential LC and regulatory LC) (Fig. 2a) and has key functional regions such as the ATP binding site, relay helix, converter region, and the LC binding domain (LCD) [9, 10].

Using the site-directed spin labeling principles developed by Hubbell and coworkers, we applied these to myosin to investigate protein structure and dynamics. SDSL was used to map the backbone dynamics and structure [14] in the N-terminus of smooth muscle myosin regulatory light chain (RLC) [13]. Structural information about the N-terminus of RLC was missing from the crystal structure of myosin, so structural models about how phosphorylation of Ser15 activates smooth muscle contraction were missing. The Thomas group designed cysteines along the N-terminus of RLC for SDSL with a 3-(5-f luoro-2,4-dinitroanilino)-PROXYL spin label (FDNASL, Fig. 1) and performed CW EPR to determine the rotational dynamics and accessibility of the probes in the presence and absence of phosphorylation. From oxygen accessibility experiments, the results revealed that in the absence of phosphorylation, the N-terminus had little or no periodicity, suggesting no secondary order; however, phosphorylation induced a strong helical pattern, internal dynamics, and accessibility, supporting a model for a disorder-to-order transition upon phosphorylation (Fig. 2b and c) [13]. This was confirmed by molecular dynamics (MD) simulations (Fig. 2d) [11, 15] and by FRET measurements that resolved two simultaneously populated structural states of RLC, closed and open, in both unphosphorylated and phosphorylated biochemical states (Fig. 2e). In addition, phosphorylation shifted the equilibrium toward the open state, increasing its mole fraction by approximately 20% [12] (Fig. 2).

## 3 Applications of Dipolar and DEER EPR: Distance Measurements

### 3.1 Myosin

**Fig. 3:**
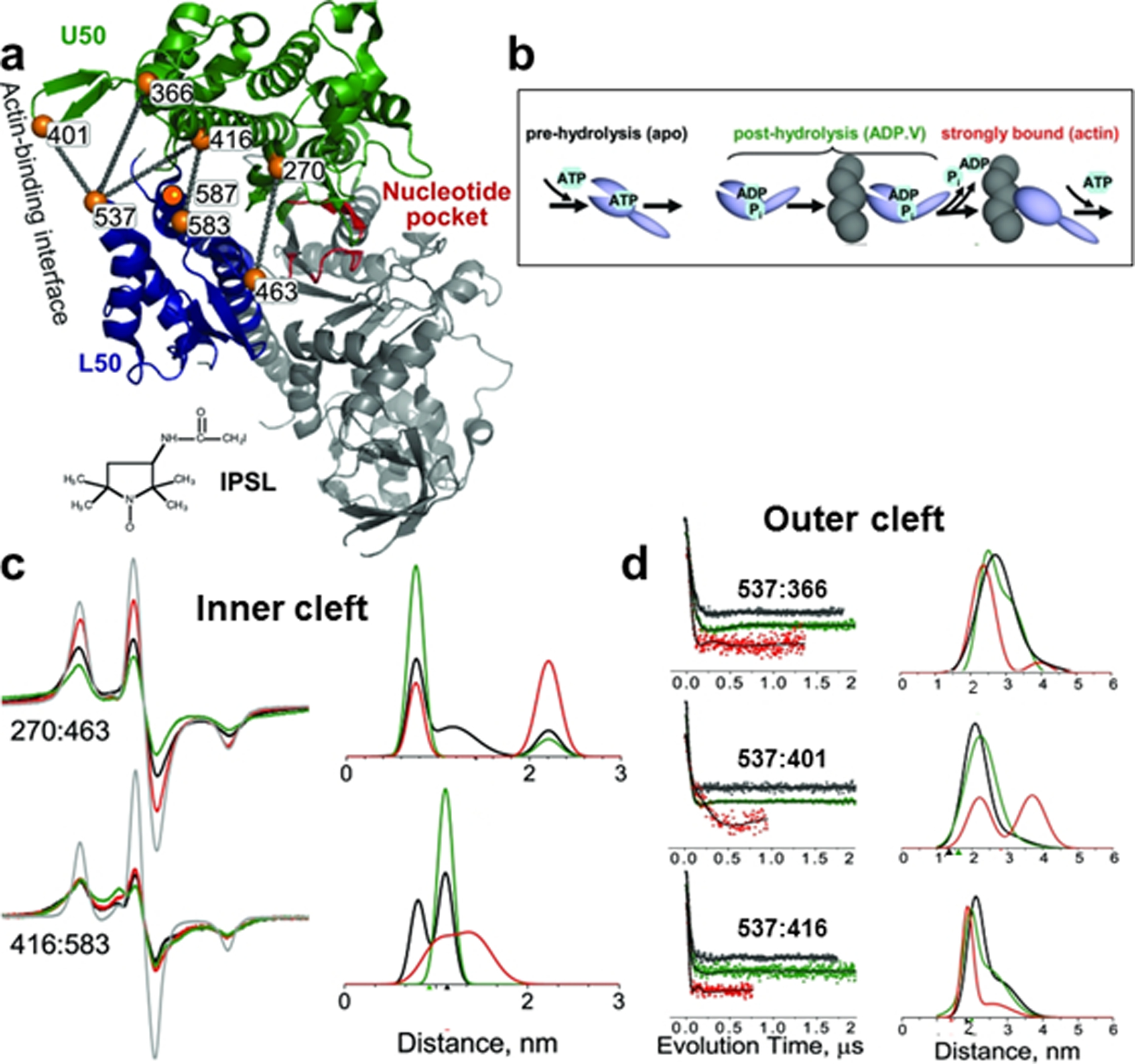
Closure of actin-binding cleft in myosin resolved by dipolar EPR and DEER. **a** Sites of Cys mutants in the actin-binding cleft of 1FMV crystal structure of *Dicty* S1dC. Lines indicate pairs used for distance measurements with IPSL-labeled myosin. **b** Simplified actin-myosin ATPase scheme showing proposed changes in the actin-binding cleft, assuming tight coupling between biochemical states. **c** Dipolar EPR in the inner cleft, *Left* Dipolar EPR spectra. A non-interacting control (single-Cys mutant, gray) and biochemical states of *apo* (pre-hydrolysis, black), ADP.V (post-hydrolysis, green), and actin-bound (red) are shown. Scan width, 200 G. *Right* Distance distributions from fits of EPR spectra, assuming a sum of multiple Gaussians. **d** DEER distance measurements in the outer cleft (color codes as in **c**). *Left* Normalized DEER decays, *Right* Distance distributions from DEER fits, assuming a sum of two Gaussians. Figure is adapted from reference with permission [16] **© (**2008) National Academy of Sciences

*Dictyostelium discoideum* (Dicty) myosin motor domain (S1dC) was an ideal protein to engineer di-cysteines at strategic positions for reaction with MTSSL because, at that time, it was the only species of myosin that was readily functionally expressed and purified for spectroscopic experiments [16]. Cysteines were designed on key helices to investigate the movements within myosin as it interacts with actin when energy from the hydrolysis of ATP is transduced into work [17]. During the work cycle, myosin alternates between states of weak and strong actin affinity, depending on the occupancy of the nucleotide-binding pocket (Fig. 3), and this is coupled to the relay helix, which translocates movements through the converter region to induce rotation of the LCD (power stroke, Fig. 3a, M*) during force generation. To repeat the cycle, myosin has to undergo a recovery stoke (Fig. 3, M**) to prime (or position) the myosin head in a pre-powerstroke state.

SDSL of engineered cysteine sites (with IPSL, Figs. 1, 3) in the actin-binding cleft of myosin combined with dipolar and pulsed EPR (DEER) to measure distances in three biochemical states: apo (pre-hydrolysis), nucleotide-bound (ADP.V) (post-hydrolysis), and actin-bound (rigor) (Fig. 3). The presence of the nucleotide analog revealed at least two distinct structural conformations in each biochemical state, indicating that the coupling between biochemical and structural states is not rigid [16]. CW dipolar EPR distance measurements of probes in the inner actin-binding cleft revealed three shorter distances in the apo state (8, 12, and 22 Å) consistent with the distances from the crystal structure, but only two distances existed in all of the nucleotide-bound (ADP.V) and actin-bound nucleotide states. Pulsed EPR (DEER) distance measurements of the probe pairs in the outer cleft were sensitive to changes in biochemical state at these sites, indicating longer distance changes in the 20–50 Å range [16]. The nucleotide-bound (ADP.V) state decreased the distance between the probes, indicating slight cleft closure, but the actin-bound state showed an even greater decrease in the distance (the shortest distance), indicating more complete closure of the cleft [16] (Fig. 3). These important findings revealed that significant myosin cleft closure occurs upon actin binding.

**Fig. 4:**
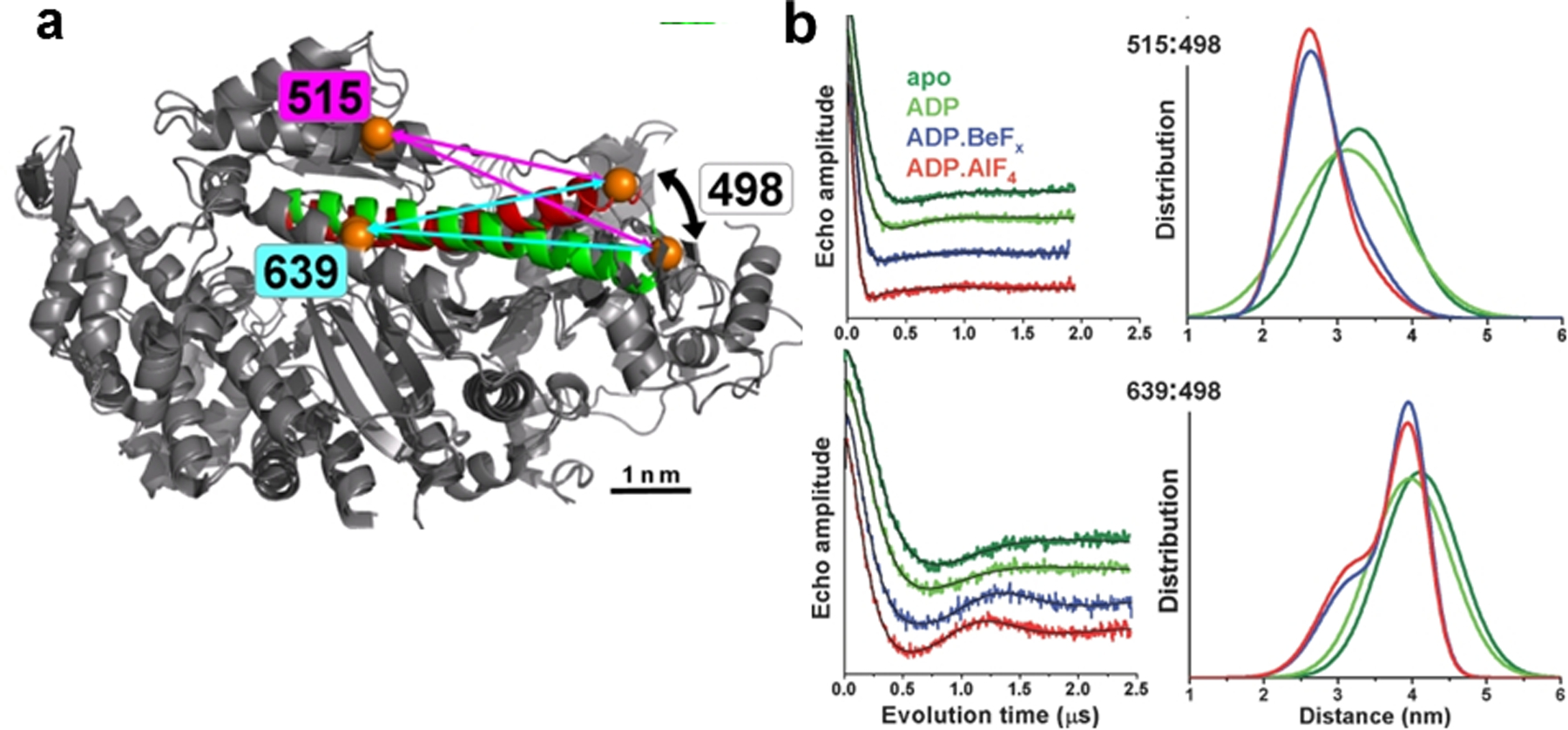
Bending of myosin relay helix resolved by DEER. **a** Myosin motor domain, overlay of crystal structures 1FMV (M*, relay helix green) and 1VOM (M**, relay helix red), showing proposed bending of the relay helix. Orange circles are labeling sites, showing predicted shortening of distance between 639 and 498 arrows) and between 515 and 498 (arrows) during the recovery stroke. **b** Bending of the relay helix resolved by DEER. (*Left*) DEER data, obtained in different biochemical states, (*right*) distance distributions from DEER data (M and M.ADP: one-Gaussian distribution, M.ADP.BeF_X_, M.ADP. AlF_4_: two-Gaussian distribution). Figure is adapted from reference with permission [18]

To investigate movements during the recovery stroke, distance measurements with TR-FRET and DEER were performed on the relay helix of myosin [18]. Based on the crystal structure of myosin, it was hypothesized that the 47 Å long relay helix couples the nucleotide-binding site to the LCD [19]. SDSL was used to attach specific probes on this helix and elsewhere on myosin (50 kd region) for TR-FRET (donor probe: IAEDANS and acceptor probe: DABCYL) and DEER (EPR probe: MSL, Fig. 1) distance measurements to elucidate the mechanism of interdomain coupling and principles of energy transduction in myosin [18]. Both methods resolved two distinct structural states of myosin, which corresponded to straight and bent conformations of the relay helix (Fig. 4). Only in the presence of nucleotides (ADP.BeF_x_ and ADP.AlF_4_) was the bent (shorter distance) state occupied, indicating that the relay helix, like the entire myosin head, bends in the recovery stroke (Fig. 4a).

### 3.2 Myosin-Binding Protein C (MyBP-C)

**Fig. 5:**
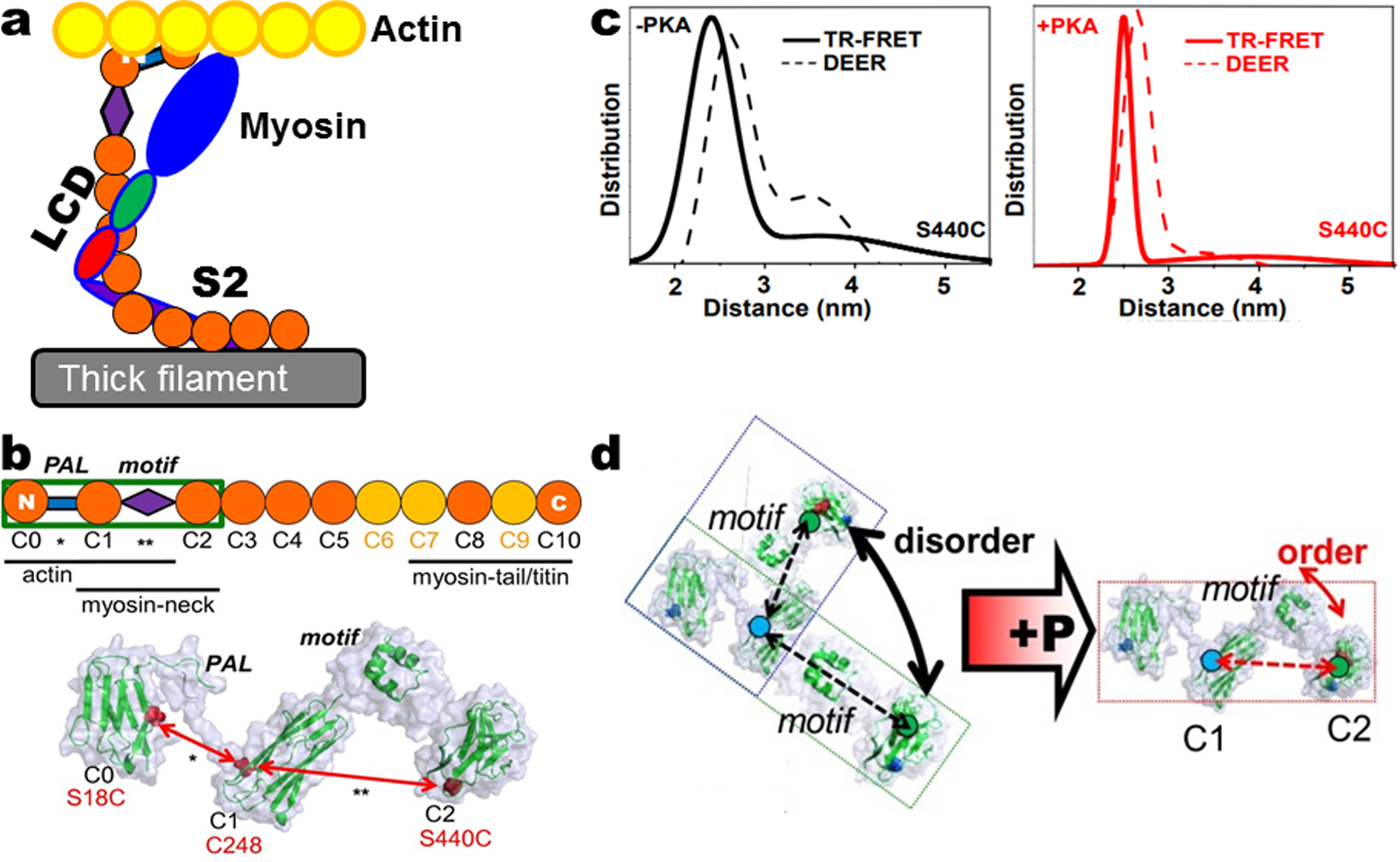
Phosphorylation-induced changes detected by DEER and FRET. **a** Model showing possible interactions of myosin-binding protein C (MyBP-C) with actin and myosin. **b** (*Top*) Cardiac MyBP-C domains (C0–C10) and proposed interaction sites with myofilament-binding proteins (*Bottom*) Proposed structure of C0C2 (in the box), showing the Ig-like domains and the intervening PAL (*) and MyBP-C motif domains (**). Site pairs (red) used to measure interdomain distances across the PAL and motif. **c** Probability distribution of distances in C0C2 (C248.S440C) motif, as determined by (*left*) TR-FRET (solid line) or DEER (dashes) in the absence of phosphorylation and in the presence of phosphorylation (*right*) by PKA using TR-FRET (red line) or DEER (red dashes). **d** Model shows phosphorylation induces open-to-closed transition in linker region of C0C2. Figure is adapted from reference with permission [20]

MyBP-C is a fairly massive protein that spans the interfilament spacing between the myosin and actin filaments. It has specific alternate binding interactions with the myosin head and actin within its N-terminal domains (C0–C1–C2 or C0C2) and anchoring binding interactions with the myosin filamentous tail at its C-terminus [21]. C0C2 contains the phosphorylation motif that regulates these binding interactions (Fig. 5). Like myosin, cardiac MyBP-C (cMyBP-C) has numerous mutations that cause hypertrophic cardiomyopathy (HCM) [22]; therefore, understanding its dynamic structural function and how HCM mutations affect it is of high importance. Using the principles of SDSL technology, intramolecular pairs of labeling sites were engineered within cMyBP-C to measure, with high resolution, the intramolecular distances and disorder in the protein’s flexible regions using TR-FRET (Donor: IAEDANS, Acceptor: FMal) and DEER (using MSL to label both cysteines) in the presence and absence of phosphorylation [20]. Distance measurements with DEER revealed that phosphorylation reduced the level of molecular disorder and that the distribution of C0C2 intramolecular distances became more compact, with probes flanking either the motif between C1 and C2 or the Pro/Ala-rich linker (PAL) between C0 and C1 (Fig. 5). Results with TR-FRET using the probes at the same sites confirmed these results [20] (Fig. 5).

### 3.3 Structural Dynamics of the Oxidation of Calmodulin (CaM)

**Fig. 6:**
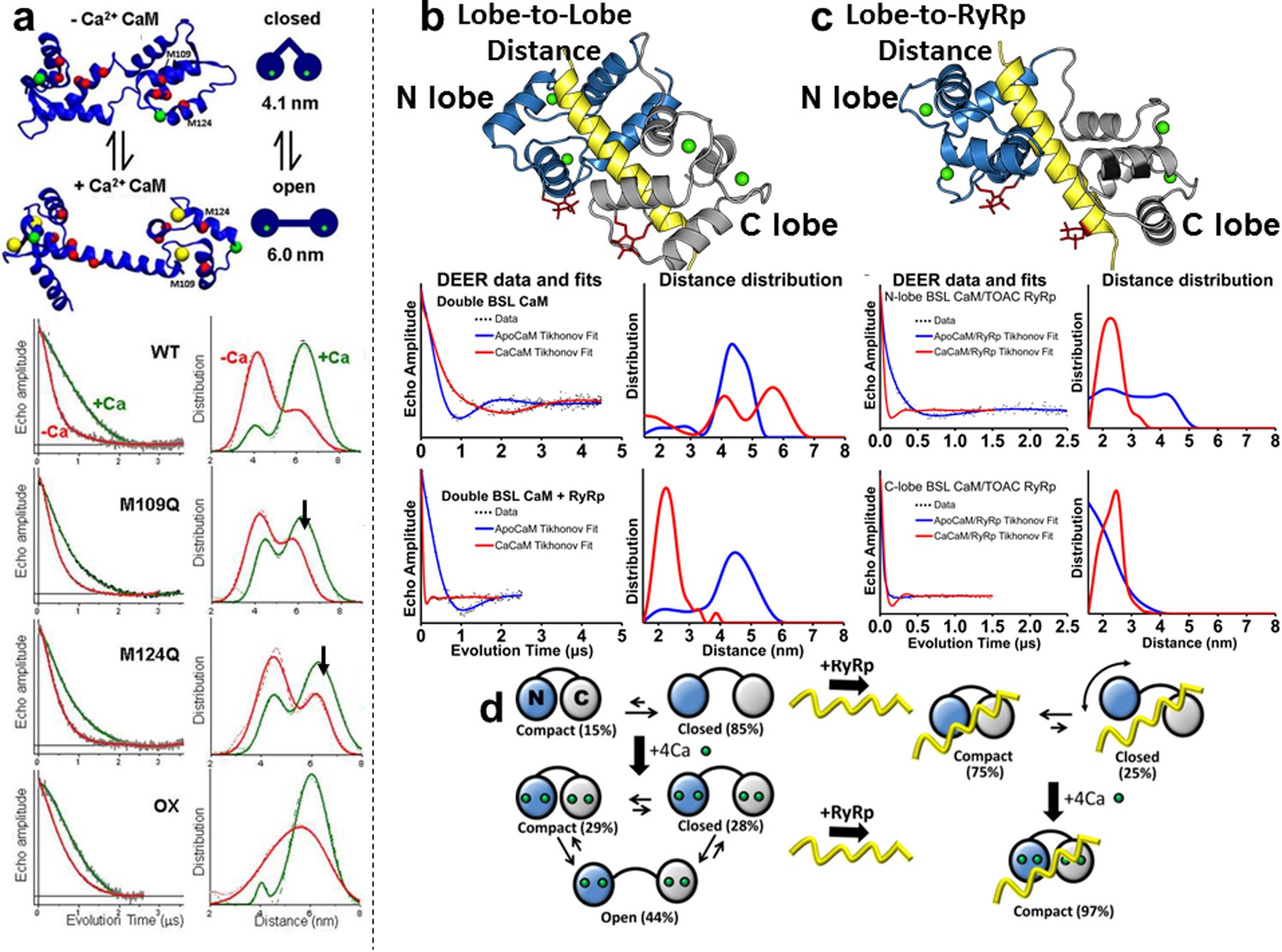
Structural dynamics of calmodulin (CaM) oxidation and interaction with ryanodine receptor RyR using DEER. **a** (*Top*) CaM structural model showing calcium ions (yellow spheres), T34C, and T117C labeling sites (green spheres), and all nine methionine residues (red spheres). (*Bottom*) DEER waveforms (*left panel*) and resulting distance distributions (*right panel*) for labeled CaM-T34C and CaM-T117C, for wildtype (WT), M109Q and M124Q mutants, and H_2_O_2_-treated WT (OX). The DEER waveforms (grey) are overlaid with the best-fit simulation (red − Ca^2+^, green + Ca^2+^). Mutations (middle panels) decrease the effect of Ca^2+^ (arrows), while oxidation of all nine methionines (lowest panel) eliminated the resolution to resolve open and closed states. **b**, **c** DEER measurements to determine interaction of CaM and a RyR peptide (RyRp). (*upper left*) model of CaM complexed with the RyR peptide (yellow) showing BSL labeling sites on CaM lobes (CaM-CaM) (*upper right*) labeling sites on lobe-to- RyRp (CaM-RyRp) for DEER measurements. (*lower panels* for **b** and **c**) DEER waveforms and Tikhonov fits (*left*) and distance distributions (*right*) for apo CaM and CaM-CaM or CaM-RyRp. **d** Model for DEER results. (*top*) In the absence of Ca^2+^ CaM is primarily in the closed structural state. Ca^2+^ binding to CaM (*lower left*) populates all three states (open, closed, compact), with the open state predominant. In the presence of RyRp and the absence of Ca^2+^ (*upper right*), the complex is in dynamic equilibrium between the compact state and a closed-like state. Ca^2+^ binding to CaM (*lower right*) shifts the complex almost completely to the compact state. Figure is adapted from references with permission [23, 24]

CaM is a Ca^2+^-binding regulatory protein that binds to several different proteins in the cell. CaM binds to the muscle calcium release channel, the ryanodine receptor (RyR) that releases Ca^2+^ from the sarcoplasmic reticulum (SR) into the cytoplasm of the muscle cell to initiate contraction [25]. Single cysteines that were introduced in the N- and C-terminal lobes of CaM for DEER measurements revealed two populations of spin labels in the presence and absence of Ca^2+^, one that indicates an open configuration where the lobes are separated by 4 nm and another closed configuration where the lobes are separated by 6 nm [24] (Fig. 6a). Ca^2+^ shifts the structural equilibrium constant toward the open state by a factor of 13. Mutations that mimic the oxidative state of CaM (M109Q and M124Q) decreased the effect of Ca^2+^, primarily by decreasing the closed-to-open equilibrium constant in the presence of Ca^2+^, suggesting that Met oxidation alters CaM’s functional interaction with its target proteins by perturbing this Ca^2+^-dependent structural shift [24] (Fig. 6a).

DEER measurements of a RyR1 (skeletal isoform) CaM-binding peptide (RyRp) labeled with a TOAC spin label complexed with CaM were performed to show that Ca^2+^-binding to the complex induces the C-terminal lobe of the RyR peptide to interact more closely with the N-terminal lobe of CaM [26]. We previously showed that TOAC-labeling of a helix results in a rigidly bound spin label [27]. Engineering di-cysteines on the N- and C-terminal lobes of CaM for labeling with BSL for a more stereospecifically bound spin label for DEER measurements revealed a third configuration of a compact CaM complexed with the RyRp [23] (Fig. 6b, c). Interprotein distance measurements showed that Ca^2+^ stabilized the compact state primarily by inducing ordered binding of the CaM N-lobe to RyRp, while only slightly affecting the C-lobe [23]. The results provided insight into the structural mechanism of CaM-mediated RyR regulation, while demonstrating the power of using two types of rigidly and stereospecifically bound spin labels (Fig. 6b–d).

### 3.4 Cytoskeletal Proteins: Utrophin, Dystrophin, and β-III-Spectrin

**Fig. 7:**
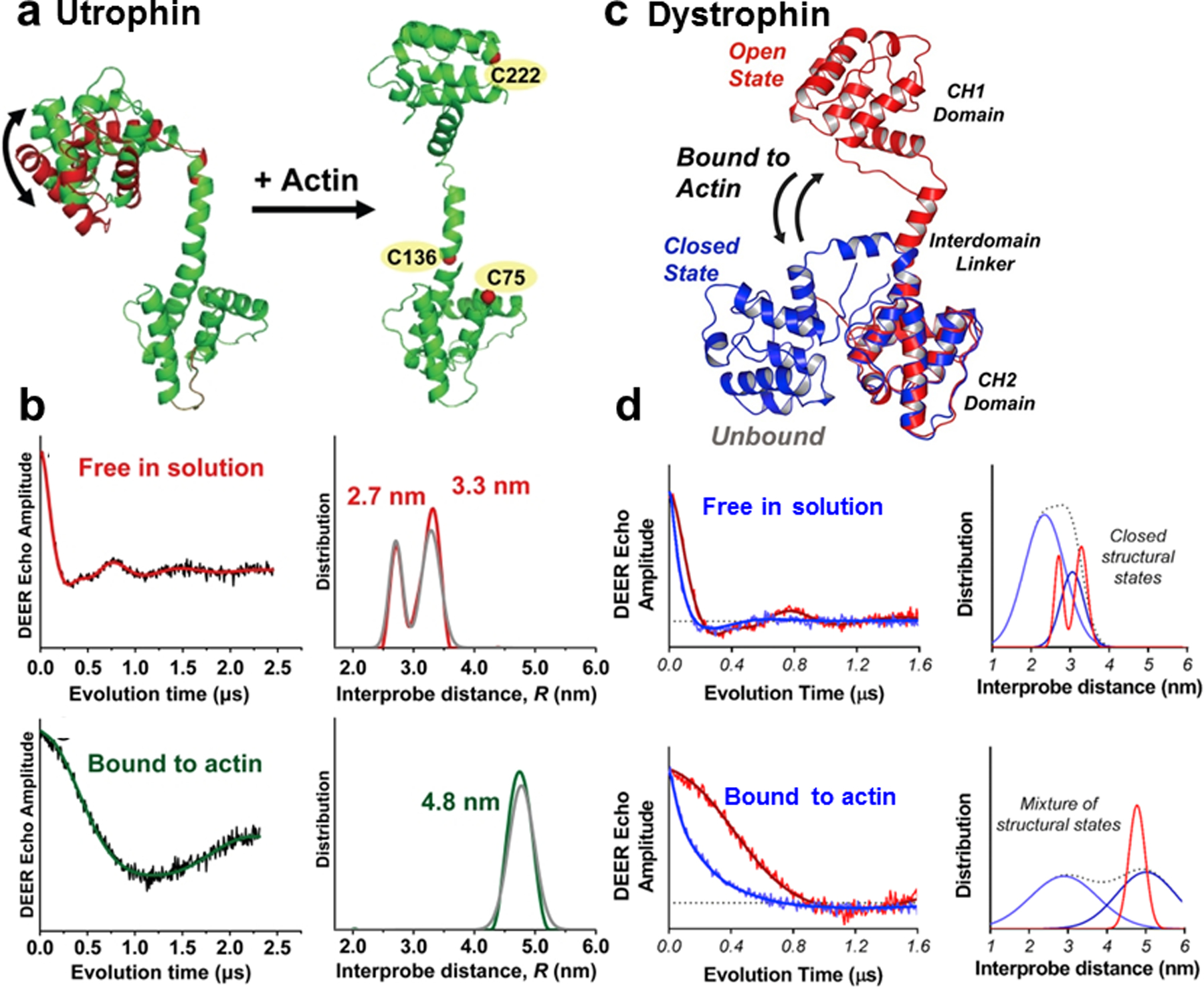
DEER measurements reveals structural dynamics of utrophin and dystrophin binding to actin. Model for utrophin (**a**) and dystrophin (**c**) binding to actin based on DEER measurements: (**a**, *left*) Two closed structures of utrophin in absence of actin, (**a**, *right*) single open structure of utrophin in presence of actin. (**c**, *left*) Single structure of dystrophin in the absence of actin (**c**, right) mixture of dystrophin structures in the presence of actin. (**b** and **d**) DEER waveforms (*left*) and distance distributions (*right*). **b** Utrophin in the absence (*upper*) and presence (*lower*) of actin. **d** Comparison of DEER (*left*) and distance distributions (*right*) of utrophin [29] and dystrophin in absence of actin (**d**, *upper*) in the presence of actin (lower). Figure is adapted from references with permission [29, 34]

Utrophin (Utr) is homolog of dystrophin (Dys), which is a large muscle cytoskeletal protein that is part of the dystroglycan complex (DGC) in the sarcolemmal membrane. Filamentous actin connects the DGC to the extracellular matrix. Utrophin is typically found in fetal or regenerating muscle but is replaced by dystrophin in the mature muscle. Utrophin has been considered to be of therapeutic potential for dystrophin disease causing mutations that cause Duchene and Becker muscular dystrophy [28]. Using SDSL and DEER, we resolved a controversy in the field based on X-ray crystallography and electron microscopy (EM) results about the interaction of utrophin and actin [29]. Crystallography showed in the absence of actin a closed configuration of Utr261 [30, 31], however, EM studies have generated controversy for Utr261, with competing closed (compact) and open (extended) models proposed in the presence of actin [31, 32]. Cysteines were engineered on the pair of N-terminal calponin homology (CH) domains in utrophin (Utr261) that are important for actin binding and labeled with MSL for dipolar DEER EPR distance measurements, which showed that the two domains are flexibly connected in solution, indicating dynamic equilibrium between two distinct structures, open and closed [29]. The open structure is consistent with the crystal structure of the domains [30]. In the presence of actin, the domains dramatically separated and became ordered, indicating a single extended and new open conformation that was not observed in the absence of actin (Fig. 7a, b). These results provided a plausible structural explanation for the decreased amplitude of actin’s torsional flexibility when the CH domains are in contact with two adjacent actin protomers [33].

A similar structural study with the dystrophin actin binding domain 1 (ABD1) and actin was conducted using DEER spectroscopy [34] to determine distances from MSL-labeled cysteine sites similar to those in the Utr261 study [29]. This study revealed that both ABDs share a common mechanism of opening upon binding to F-actin, however, the Dys ABD1 contains much more structural disorder than seen previously in Utr ABD1 and unlike the Utr ABD1, Dys ABD1 does not undergo a complete shift to the open structural state [34] (Fig. 7c, d). Therefore, collectively, the pulsed EPR data identified a common binding mode for Dys and Utr ABD1, but simultaneously highlighted distinct structural dynamics in the ABD–actin interaction.

**Fig. 8:**
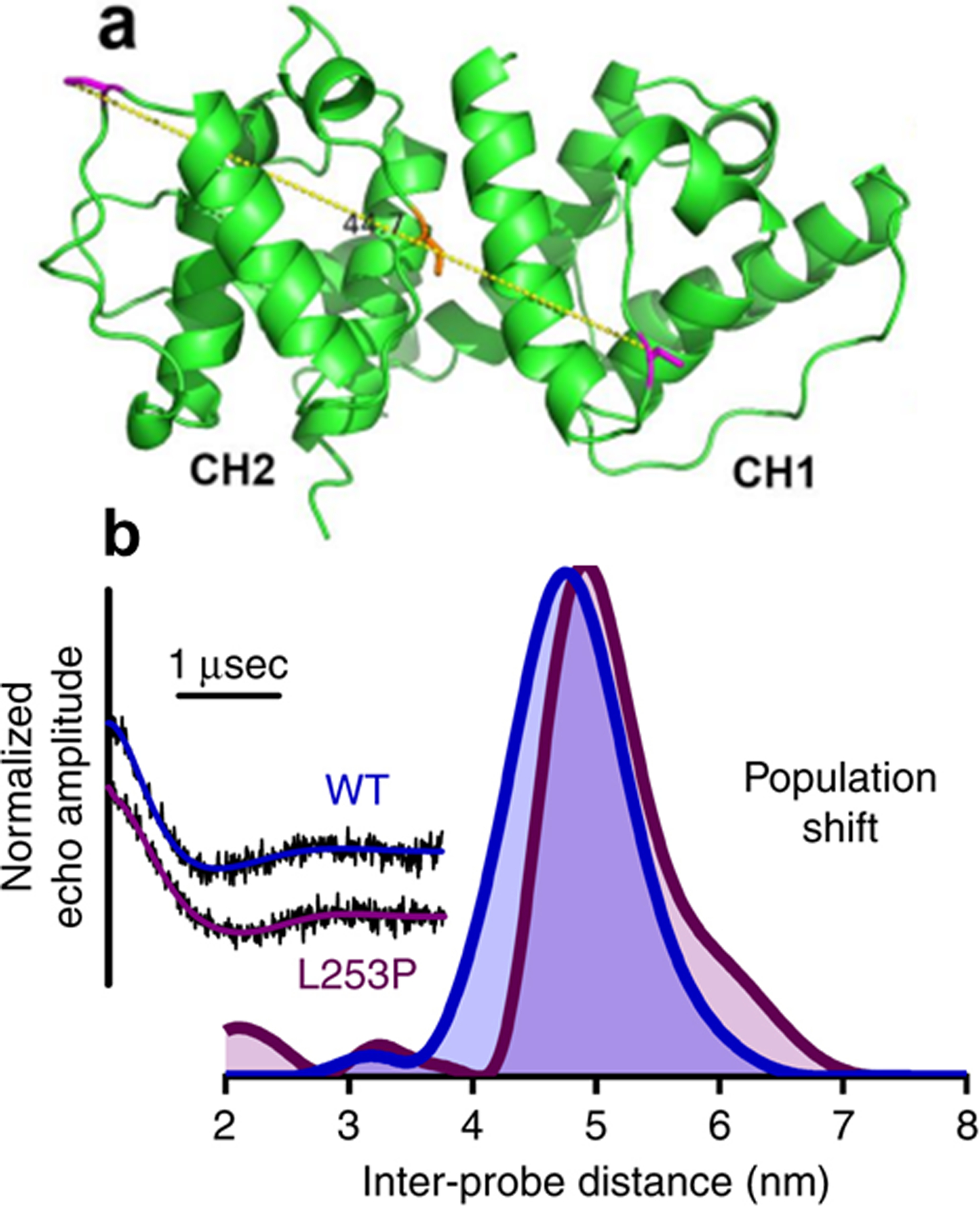
Structural mechanism for high-affinity actin binding by a β-III-spectrin SCA5 missense mutation is revealed by DEER measurements. **a** Homology model generated for β-III-spectrin ABD from crystal structure of the closed state of α-actinin, showing the approximate inter-probe distance (side chainside chain distance of 4.5 nm, yellow dotted line) between two native cysteine residues (C76 and C231, magenta residues) and highlighting L253 in orange at the CH domain interface. **b** DEER measurement showing the L253P mutation opens the β-III-spectrin ABD structure. Echo amplitude decays of WT ABD (blue) and L253P ABD (purple) along with their corresponding Tikhonov fits are shown on the left. The inter-probe distances derived from Tikhonov regularization for both WT and L253P ABDs are shown on the right. The WT ABD distance distribution is centered at 4.8 nm, consistent with the distance predicted in the homology model of the closed state. Upon introduction of the L253P mutation, the distance distribution undergoes a shift to populate a longer inter-probe distance, visible as a shoulder to the right of the 4.8 nm peak, consistent with structural opening of the ABD. Figure is adapted from reference with permission [39]

β-III-Spectrin is a cytoskeletal protein that is required for normal dendrite structure and synaptic transmission for normal cerebellar control of motor coordination [35]. Autosomal dominant mutations in β-III-spectrin of Purkinje cells cause a neurodegenerative disease called spinocerebellar ataxia type 5 (SCA5) [36, 37]. β-III-Spectrin has an actin-binding domain (ABD) consisting of tandem calponin homology (CH) domains. A SCA missense mutation, L253P, is localized to the second subdomain (CH2) and caused a 1000-fold increase in actin binding [38]. To elucidate the structural mechanism of L253P mutation, SDSL of engineered MSL-labeled cysteines in the two CH domains of β-III-spectrin containing the L253P mutation were used for distance measurements with DEER spectroscopy in the presence and absence of actin [39] (Fig. 8). Modeling of the results indicated that the L253P mutation of β-III-spectrin induced high-affinity actin binding by disrupting a regulatory mechanism that shifts the ABD structural equilibrium from a closed to more open binding-competent state [39] (Fig. 8).

### 3.5 Aurora Kinase

Aurora A (AurA) is a member of the serine/threonine protease kinases that play a central role in mitosis [40]. It exists in distinct pools that are defined by its function in protein–protein interactions and posttranslational modifications such as in controlling centrosome maturation and mitotic entry or nucleation of microtubules by chromatin and construction of the bipolar mitotic spindle [41]. AurA is activated by two distinct activation mechanisms operating in different spatiotemporal contexts: (1) autophosphorylation of T288 for centrosomal functions and (2) activation by a spindle assembly factor, Tpx2, at the mitotic spindle [41]. The phosphorylation site is within the activation loop that contains a conserved asp-phe-gly (DFG) motif at the N-terminal, which adopts a DFG-In conformation in the active state and a DFG-Out conformation in the inactive state [42].

**Fig. 9:**
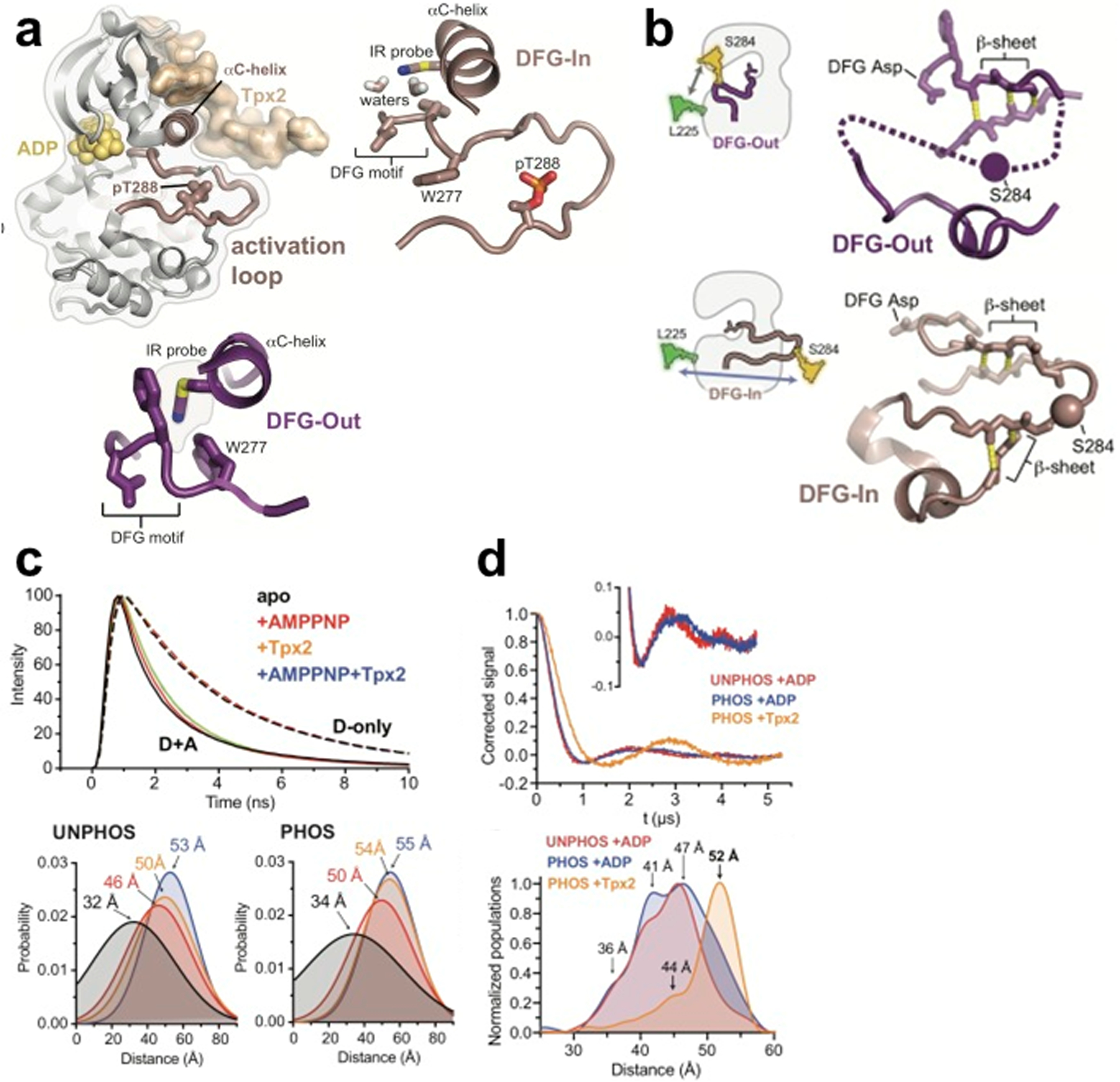
DEER and FRET distance measurements resolve dynamics in allosteric activation of Aurora kinase A (AurA) by activation loop phosphorylation. **a** Structure of AurA (*left*) in the active conformation bound to ADP (yellow) and Tpx2 (beige), with enlarged views of the DFG-In (*right*) and DFG-Out (*lower*) states. The phosphorylated activation loop remains flexible and shifts to a more active conformation upon Tpx2 binding. **b** Schematics showing the labeling scheme used to detect the DFG-In/Out transition by FRET and DEER: structures of the DFG-Out (top) and DFG-In (bottom) states of AurA. **c** (*Upper*) Time-resolved fluorescence waveforms for D-only (dashed lines) and D + A (solid lines) phosphorylated AurA in the presence and absence of 125 μM Tpx2 and 1 mM AMPPNP. (*Lower*) Comparison of single-Gaussian distance distribution fits to fluorescence lifetime data obtained with unphosphorylated (left) and phosphorylated AurA (right). **d** DEER spectroscopy confirms that phosphorylation of AurA alters the DFG-In state. (*upper left*) DEER waveforms of unphosphorylated AurA bound to ADP (red), and phosphorylated AurA bound to either ADP (blue) or to Tpx2 (yellow). The inset shows enlarged view with + ADP. (*lower*) Population densities from Tikhonov analysis of waveforms in upper panels. Figure is adapted from reference with permission [43]

The Thomas group collaborated with the Levinson group to apply FRET and DEER spectroscopy to determine the effects of phosphorylation on the structure of the DFG-In and DFG-Out states [43] (Fig. 9a, b). Time-resolved (TR) FRET measurements of probes in the activator loop and in an adjacent helix (Fig. 9b) showed that the distance distributions measured for the phosphorylated and unphosphorylated kinase in the absence of ligands were strikingly similar with broad distributions centered at 30 Å [43]. Therefore, FRET did not resolve the mechanism of how phosphorylation affected the kinase switching between active and inactive states. Subsequent, molecular dynamics simulations revealed that the activation loop was not disordered but existed in two discrete populations (Fig. 9c). DEER experiments with probes (MTSSL) in the same location as FRET probes confirmed the broad distribution of distances spanning 30–55 Å, suggesting multiple conformations. Careful analysis of the DEER spectra indicated that distances beyond 50 Å were more populated in the phosphorylated sample, suggesting that by eliminating the autoinhibited DFG-In substate, phosphorylation redistributes the ensemble between the DFG-Out and active DFG-In substates. Additionally, in the presence of Tpx2, the DEER spectra of phosphorylated AurA revealed a single dominant peak at 52 Å (not observed in the absence of Tpx2), which is consistent with the activation loop adopting the extended DFG-In conformation [43]. In summary, it appears that phosphorylation and Tpx2 activate AurA through very different but complementary mechanisms, with phosphorylation triggering a structural switch within the DFG-In subpopulation, and Tpx2 instead promoting a DFG flip to increase the population of the DFG-In state, confirming the higher resolution of DEER spectroscopy experiments (Fig. 9d).

**Fig. 10:**
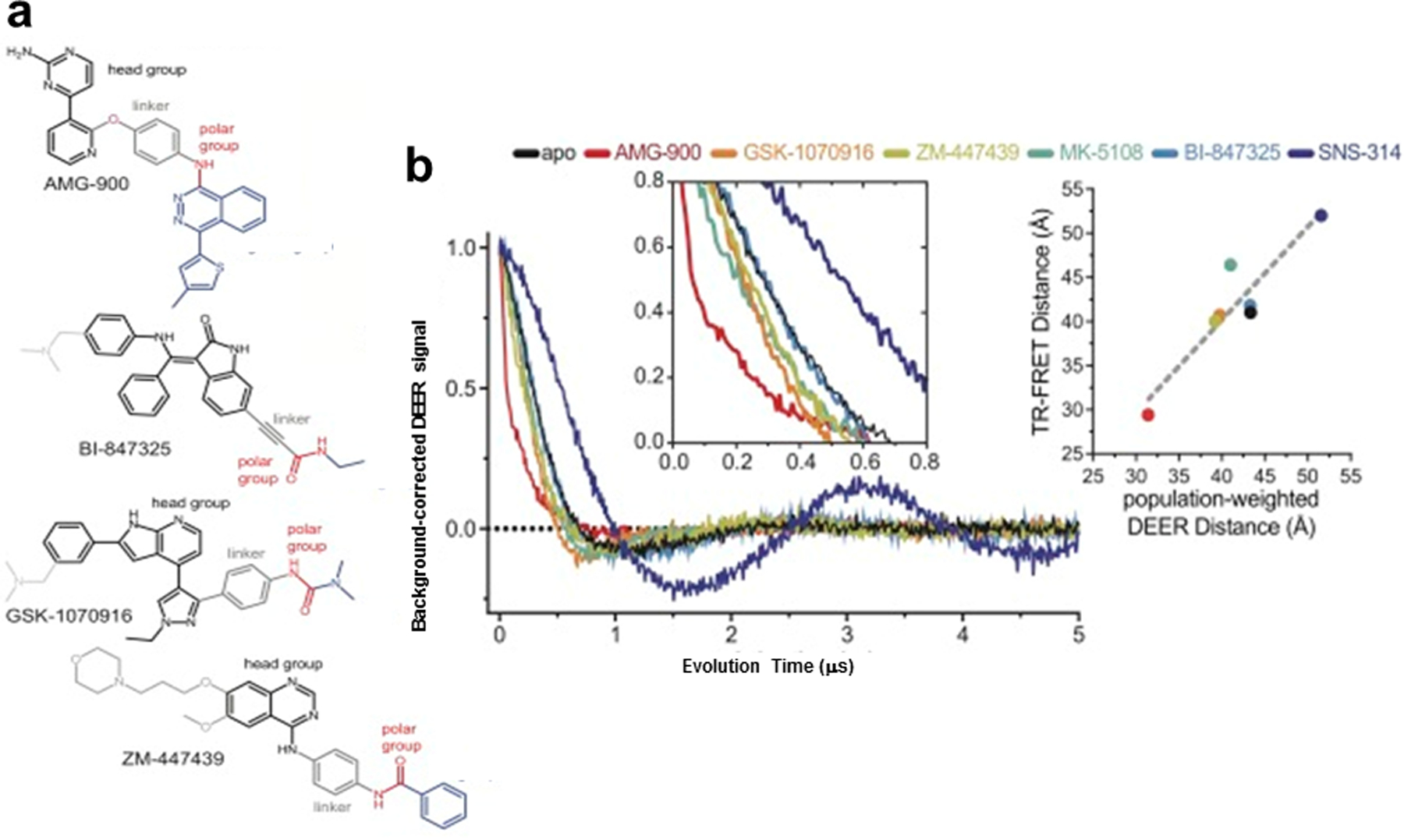
Inhibitors that induce a complete switch to the DFG-out state. **a** Chemical structures of the type II DFG-out compounds. **b** DEER spectra of phosphorylated AurA bound to each of the four putative type II inhibitors, MK-5108 or SNS-314. The *left inset* shows an expanded view of the echo decays at short evolution time, which reflect the average spin–spin distances. A comparison of the population-weighted average DEER Tikhonov-derived distances were plotted against the corresponding TR-FRET distances in the *right inset*. Figure is adapted from reference with permission [45]

AurA also performs other important cellular functions through the formation of complexes with regulatory proteins, including the oncogenic transcription factors c-Myc and N-Myc, and is amplified in a variety of solid tumors [44]. This motivated the search for Aurora inhibitors, many of which are in clinical trials, but as of yet none have received regulatory approval. In a parallel collaboration, Thomas–Levinson sought to study the conformational effects of Aurora kinase inhibitors across different activation states of AurA and showed that all inhibitors, including type I and type II compounds, caused substantial structural changes upon binding, and that these effects influenced binding selectivity [45] (Fig. 10a). They used TR-FRET to determine the effects of 24 kinase inhibitors on four biochemical states of AurA and found a wide range of conformational preferences, with all inhibitors promoting either the active DFG-in state or the inactive DFG-out state, but to widely differing extents (Fig. 10b). Six of the kinase inhibitors were tested with DEER experiments using the same AurA cysteine sites to label with probes and they found results complementary to the TR-FRET results [45] (Fig. 10b).

## 4 Application of SDSL: Bifunctional Spin Labels

### 4.1 Myosin

**Fig. 11:**
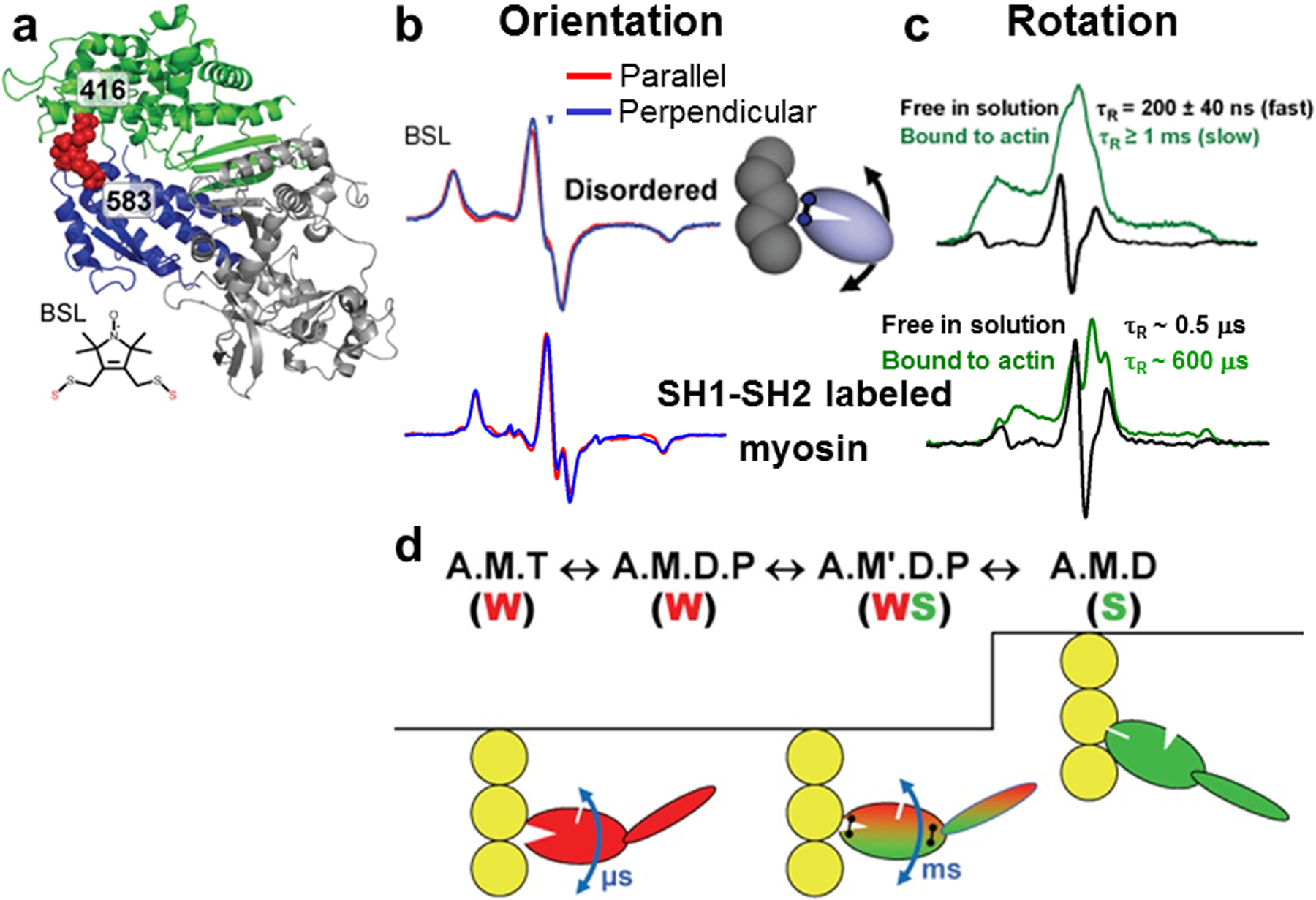
Rotational motions of myosin measured with BSL. **a** Crystal structure of myosin head BSL-crosslinked at positions 416 and 583. **b** Conventional EPR spectra of labeled S1 decorated fiber bundles parallel or perpendicular to the magnetic field **c** ST-EPR spectra showing rotational dynamics of labeled S1 in solution (black) or bound to actin (green). Top: BSL-labeled myosin head. **Bottom:** SH1-SH2 labeled myosin S1. **d** Model showing myosin head dynamics coupled to a simplified ATPase scheme in which the actin-binding cleft of myosin transitions from an open structural state having a dynamically (μs rotational correlation time (τ_R_) disordered catalytic domain when weakly bound (W, *left*) to a closed state having an immobile and ordered catalytic domain (S, *right*). The *black linkers* shown in the WS state represent two cross-linking sites, across the actin-binding cleft or between SH1 and SH2. Figure is adapted from references with permission [52, 53]

The Thomas lab have used the high-resolution power of CW EPR to measure the changes in orientation of myosin in muscle fibers [46–51]. The global orientation of proteins was measured under physiological conditions using monofunctional spin labels that enabled rigid and stereospecific labeling. However, the labeling sites were limited unless engineered on smaller proteins and exchanged into muscle fiber bundles. Despite being able to mutate, express, and purify Dicty myosin, the study of myosin orientation was limited to decorating fibers with the myosin fragment and study actin-bound orientations (rigor and complexed with ADP), i.e., in the absence of ATP, which dissociates the myosin fragment from actin. Nevertheless, this enabled the engineering of cysteines for SDSL with monofunctional spin labels and bifunctional spin labels (BSL) to measure rotational motions of myosin S1 with STEPR [52, 53] in the presence and absence of actin (Fig. 11) and orientation changes in myosin segments [54–56] and in the LCD in muscle fibers [57]. BSL labeling adjacent cysteines acts like a crosslinker and was effective in cross-linking the actinbinding cleft of myosin [53] and SH1-SH2 cysteines in myosin [52] to capture elusive intermediate states (Fig. 11) [52, 53].

**Fig. 12:**
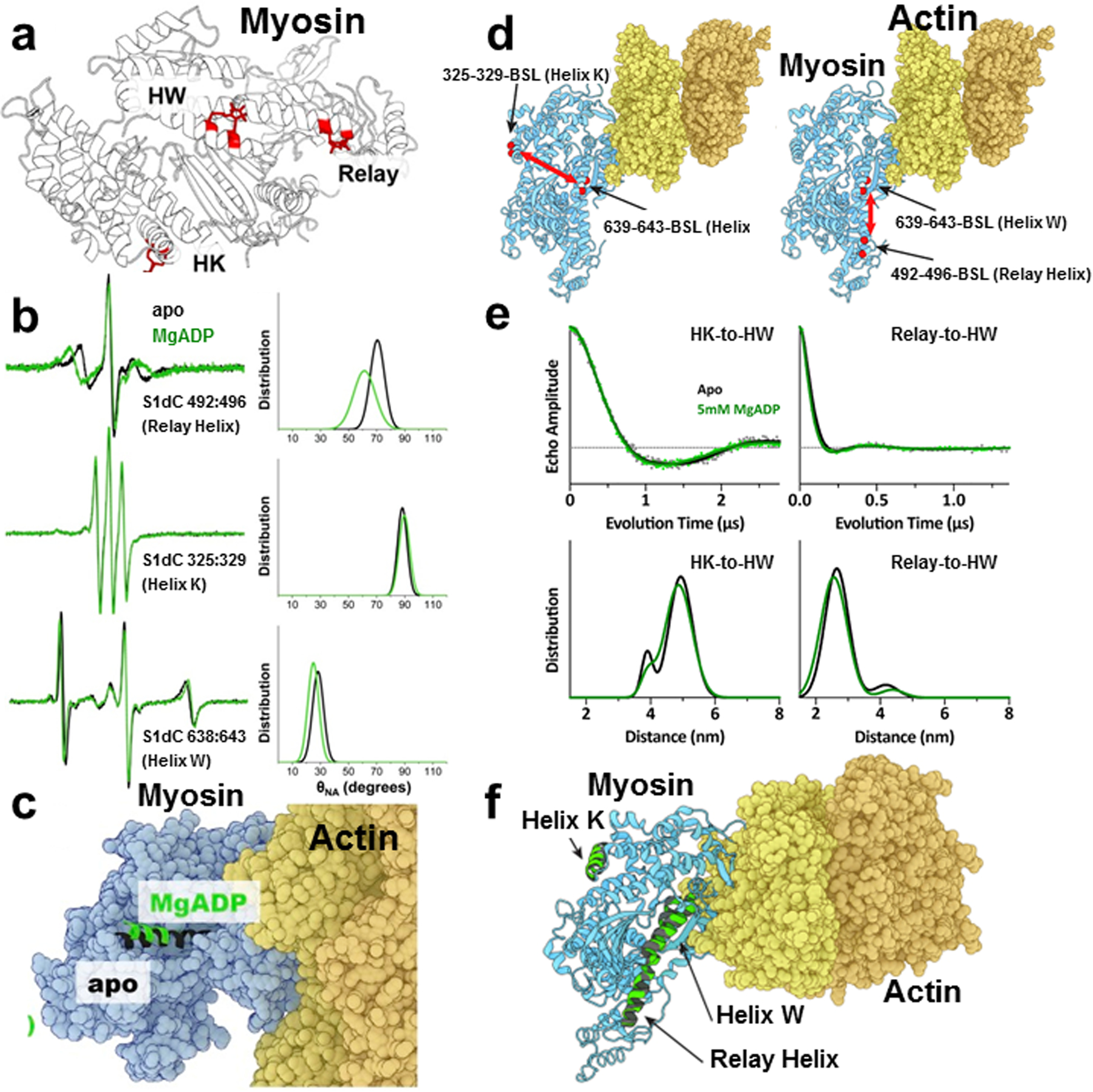
Orientation and distance constraints using BSL-labeled myosin. *Left column*: **a**–**c** Orientational changes in BSL-labeled Dicty myosin S1 (S1dC) using CW EPR. **a** BSL modeled into myosin head on Helix W, Helix K, and the Relay Helix. **b** CW EPR spectra of apo myosin (black) and MgADP-bound S1dC-actin complex (green). **c** Actin in complex with myosin showing the relay helix in apo (black) and MgADP (green) myosin from modeling orientational changes. *Right column* (**d**, **e**) Distance measurements in BSL-myosin using DEER. **d** Actin–myosin complex showing the labeling sites for BSL. **e** Distance measurements with DEER in apo and MgADP-bound S1dC-actin complex. **f** Actin–myosin complex showing the helices in the apo and MgADP-bound myosin from modeling of measured distances. Figure is adapted from references with permission [54, 56]

Di-cysteines were engineered on known α-helical segments on the catalytic domain of Dicty myosin for attachment of BSL and exchanged into rigor fiber bundles to study the orientation of the spin labels in the absence of nucleotides and in the presence of ADP [54, 55] (Fig. 12). ADP affinity for myosin is decreased by a factor of 100 by actin, and strain on the LCD affects the rate of ADP release in many myosins. In smooth myosin ADP, release is associated with LCD movement, so understanding the allosteric coupling between the nucleotide-binding pocket and the actin-binding interface is important [58]. They observed reversible rotations of two helices (in the relay region) in the actin-bound myosin in response ADP binding and dissociation [54] (Fig. 12a–c). This orientation data was later combined with distance constraints from DEER experiments with BSL attached in same region to model the structure of myosin-II when bound to actin filaments [56] (Fig. 12d–f).

### 4.2 Phospholamban (PLB)

PLB is a small regulatory peptide of the sarco/endoplasmic reticulum calcium ATPase (SERCA) membrane protein in the heart. SERCA uses Ca^2+^-dependent hydrolysis of ATP to fuel active transport (uptake) of cytosolic Ca^2+^ into the SR or ER. PLB regulates this process through phosphorylation to change the affinity of PLB for SERCA [59].

**Fig. 13:**
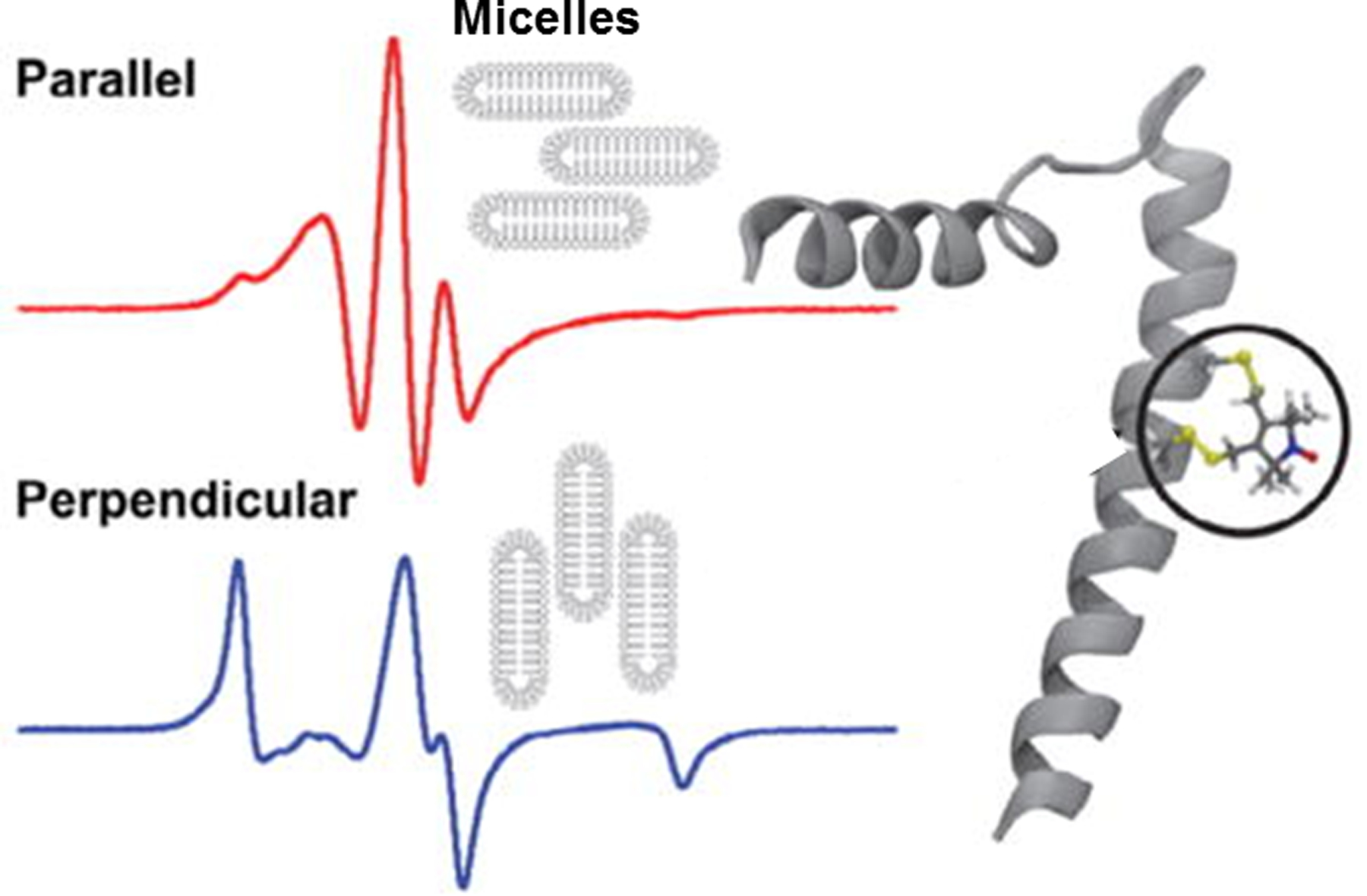
BSL reports on the structural topology of phospholamban (PLB) in magnetically-aligned bicelles. EPR spectra (t*op*, *left*) of bifunctional 32/36-BSL-PLB (*right*) in bicelles with the membrane normal aligned parallel (top) and perpendicular (bottom) with respect to the applied magnetic field. Spectral analysis yielded a label tilt angle of 90° ± 3°. Spectra were acquired at 298 K. The sweep width on horizontal axis is 120 Gauss. Figure is adapted from reference with permission [60]

To assess the structural topology of PLB in magnetically aligned bicelles, single cysteine and di-cysteines PLB mutants were created for SDSL with a monofunctional and bifunctional spin label (BSL) [60]. BSL attached rigidly and stereospecifically to PLB, in contrast to the monofunctional spin labels, which showed higher mobility. BSL-PLB reported similar orientation in the bicelles as the rigid and stereospecific TOAC label that was bound directly to the backbone of PLB via chemical synthesis [60]. The EPR spectra of BSL-PLB obtained from the bicelles aligned parallel or perpendicular to the magnetic field showed distinct differences throughout the EPR line shape that are indicative of highly oriented spin labels with a narrow well-defined orientation and distribution (Fig. 13). In contrast, the EPR spectrum of monofunctional spin-labeled PLB shows broadened features at low and high field when the bicelles were parallel to the magnetic field, but in the perpendicular orientation, the spectrum had significantly broadened throughout, indicating orientation disorder and/or sub-microsecond rotational dynamics. Simulating and fitting the BSL spectra yielded a tilt angle of 90° ± 3° relative to the magnetic field axis, which was consistent with MD simulations of PLB that revealed a tilt angle of 89° ± 4° (Fig. 13). Thus, BSL provided a comparable alternative to TOAC where peptide synthesis is required and expands the field to provide accurate information about orientation and rotational dynamics of structural elements in membranes.

## 5 Conclusions and Future Perspectives

Significant advances in EPR technology have brought this biophysical technique to the forefront for studying structural dynamics that are complementary to FRET spectroscopy. The pioneering SDSL work of Wayne Hubbell and collaborators made both dipolar EPR and DEER distance measurements in macromolecular assemblies possible to study dynamic interactions that were not detected by other structural biophysical techniques. The Thomas group and collaborators have applied these techniques successfully in numerous collaborations to elucidate the structural dynamics of diverse proteins, while also extending EPR technology to the microsecond time range, through the development of STEPR, as needed for the study of large protein complexes or viscous environments [61]. Our group has also taken advantage of the higher throughput of fluorescence spectroscopy to applications in drug discovery, using our novel fluorescence lifetime microplate reader for high-throughput screening (HTS) of large compound libraries [62–64]. Future advances in EPR technology would benefit from innovative exploration to develop EPR spectroscopy for medium to high-throughput detection.

## Data Availability

Data sharing not applicable to this review article as no datasets were generated or analyzed during the current study. All the data presented in this review article have been previously published and are cited in the paper.
